# Epidemiology of inflammatory bowel disease in Mexico and Colombia: Analysis of health databases, mathematical modelling and a case-series study

**DOI:** 10.1371/journal.pone.0228256

**Published:** 2020-01-27

**Authors:** Agustín Ciapponi, Sacha Alexis Virgilio, Mabel Berrueta, Natalie Claire Soto, Álvaro Ciganda, Moisés Freddy Rojas Illanes, Briseida Rubio Martinez, Johana Gamba, Carlos Arturo González Salazar, José Nicolás Rocha Rodríguez, Bruno Scarpellini, Ana María Bravo Perdomo, Gerardo Machnicki, Leandro Aldunate, Juan De Paula, Ariel Bardach

**Affiliations:** 1 Instituto de Efectividad Clínica y Sanitaria (IECS), Buenos Aires, Argentina; 2 Hospital Dr. Bernardo Sepúlveda, Mexico DF, Mexico; 3 Fundación Universitaria Sánitas, Bogotá, Colombia; 4 Janssen-Cilag Farmacêutica Ltda, São Paulo, Brazil; 5 Janssen-Cilag Farmacêutica Ltda, Bogotá, Colombia; 6 Janssen-Cilag Farmacêutica Ltda, Buenos Aires, Argentina; 7 Hospital Italiano de Buenos Aires (HIBA), Buenos Aires, Argentina; Center for Primary Care and Public Health, SWITZERLAND

## Abstract

**Background and aims:**

Ulcerative Colitis (UC) and Crohn's Disease (CD) have a major impact on quality of life and medical costs. The aim of the study was to estimate the prevalence, incidence and clinical phenotypes of Inflammatory Bowel Disease (IBD) cases in Mexico and Colombia.

**Methods:**

We analyzed official administrative and health databases, used mathematical modelling to estimate the incidence and complete prevalence, and performed a case-series of IBD patients at a referral center both in Mexico and Colombia.

**Results:**

The age-adjusted complete prevalence of UC per 100,000 inhabitants for 2015/2016 ranged from 15.65 to 71.19 in Mexico and from 27.40 to 69.97 in Colombia depending on the model considered. The prevalence of CD per 100,000 inhabitants in Mexico ranged from 15.45 to 18.08 and from 16.75 to 18.43 in Colombia.

In Mexico, the age-adjusted incidence of UC per 100,000 inhabitants per year ranged from 0.90 to 2.30, and from 0.55 to 2.33 in Colombia. The incidence for CD in Mexico ranged from 0.35 to 0.66 whereas in Colombia, the age-adjusted incidence of CD ranged from 0.30 to 0.57.

The case-series included 200 IBD patients from Mexico and 204 patients from Colombia. The UC/CD prevalence ratio in Mexico and Colombia was 1.50:1 and 4.5:1 respectively. In Mexico, the female/male prevalence ratio for UC was 1.50:1 and 1.28:1 for CD, while in Colombia this ratio was 0.68:1 for UC and 0.8:1 for CD. In Mexico the relapse rate for UC was 63.3% and 72.5% for CD, while those rates in Colombia were 58.2% for UC and 58.3% for CD.

**Conclusions:**

The estimated burden of disease of IBD in Mexico and Colombia is not negligible. Although these findings need to be confirmed by population-based studies, they are useful for decision-makers, practitioners and patients with this condition.

## Introduction

Inflammatory bowel disease (IBD) is a term encompassing two main inflammatory diseases of the colon and/ or small intestine: Crohn's disease (CD) and Ulcerative Colitis (UC).[1] Diagnosis is based on clinical presentation, endoscopic findings and other imaging and histopathologic findings. Both diseases are chronic and clinically intermittent with remissions and relapses, possibly due to interactions between genetic and environmental factors.[2] Differentiation between UC and CD is not always clear as symptoms overlap and extra-intestinal manifestations can be similar. Treatment of inflammatory bowel disease includes lifestyle changes (e.g., smoking cessation for patients with CD), medical management, and surgical interventions.[1]

IBDs have a major impact on life expectancy, quality of life and medical costs. For example, patients with Crohn’s disease have a risk of dying over 50% higher than someone in the general population of the same age.[3] IBD burden derives in an important increase of direct medical costs, although this may vary across countries. A Canadian study showed that IBD doubles healthy controls direct costs and that CD is on average 20% costlier than UC.[4]

Regarding UC, the cause of the disease is unknown; however, over‐stimulation of an inadequately regulated mucosal immune system appears to be a major pathophysiological pathway. The course of the disease is generally relapsing–remitting, with patients experiencing few or no gastrointestinal symptoms in between symptomatic flare‐ups (relapses).

The prevalence of UC in Europe and USA reaches 70–150 patients per 100,000 inhabitants.[5] The overall mortality rates for CD have decreased around the world in the last twenty years, but based on recently published nationwide data there is still excess mortality during the disease course.[6,7] Mortality rates in CD vary in different regions of the world, that may be related with different genetic, environmental, and health care related conditions.[6,7] IBD’s epidemiology is well characterized in developed countries, but this is less so in Latin America (LA). However, a study reports that since 1990 IBD incidence has been rising in newly industrialized countries in South America and other regions[8]. A systematic review including LA reported considerably lower prevalence rates for said region than for others[9] and marked differences in the burden of the disease among countries. Environmental factors such as socio-economic status, exposure to infections, use of antibiotics and issues of hygiene, might help explain the epidemiological differences between populations.[10]

Some studies have attempted descriptions of the epidemiology of IBD in Latin America.[11–18] UC incidences ranged from 0.74 to 6.76 per 100,000 inhabitants, 0.24 to 3.50 for CD, and 0.42 to 2.46 for non-specified IBD. UC prevalence ranged from 0.99 to 44.3 per 100,000 inhabitants, 0.24 to 14.90 for CD and 0.42 to 38.22 for non-specified IBD.[18] Some case-series and descriptive studies also describe some features of these conditions in patients of LA. [19–24]

Despite the little evidence from the IBD patients in Latin America, our hypothesis is that the burden of disease in LA is not marginal. Considering this panorama of scarcity of epidemiological, clinical and resource use information, we aimed at conducting a study to estimate the prevalence, incidence and clinical phenotypes of IBD in Mexico and Colombia, two large countries of the region for which data is also lacking.

## Materials and methods

We performed a retrospective observational study following the *Strengthening The Reporting of Observational Studies in Epidemiology* (STROBE) guidelines.[25] During 2018, we included data from official administrative and health databases in Mexico and Colombia. We used the tenth revision of the International Classification of Diseases (ICD 10) IBD, selecting codes K50x (for CD) and K51x (for UC). We also explored related ICD codes, as K564: Other obstructions of the intestine, K63 Other diseases of the intestines, K638 Other specified diseases of the intestine, K639 Intestinal disease, unspecified, K591 Functional diarrhea, M075 Joint disease in ulcerative colitis, M095 Juvenile arthritis in ulcerative colitis and K529 Colitis and gastroenteritis not infectious, not specified. However, we decided not to include these additional codes because of their very low specificity. Because the capture-recapture approach requires the inclusion of health facilities serving at least 80% of the population from a geographic area to estimate the target disease epidemiology with reasonable reliability, and this type of centers does not exist in Mexico and Colombia, we had to resort to alternative methods. We therefore analyzed official administrative databases, modelling incidence and prevalence with mathematical models.

### Mexico data

As part of the process of estimation of prevalence and incidence of IBD cases in Mexico, which relate to the true burden of disease, we initially analyzed databases with information on hospital discharges by IBD, sex and age group generated by the Mexican public health sector.

Public sector discharge records were obtained from a standard-format database of hospital discharges in the health sector (2009–2015), which is available on the website of the National Health Information System (Sistema Nacional de Información en Salud—SINAIS) of the General Directorate of Health Information (Dirección General de Información en Salud—DGIS).[26] This database includes more than 40 million hospital discharge records during the evaluated period and provides basic information such as year of care, sex, age of patient, and main condition. The records are coded according to the ICD-10,[27] length of stay, reason for discharge, and hospital discharges from the care institution, and have been compiled by the Ministry of Health, the Mexican Social Security Institute (Instituto Mexicano del Seguro Social—IMSS), Oportunidades IMSS, the Institute for Social Security and Services for State Workers (Instituto de Seguridad Social y Servicios para Trabajadores del Estado—ISSSTE), PEMEX and the Mexican Navy (SEMAR). According to the Official Mexican Standard, hospital discharges information includes number of discharges due to cure, improvement, transfer to another hospital unit, death or discharge, and excludes transfers between different services within the hospital.[28] It is important to consider that while hospital discharge records compile data for each event, a person can be admitted to the hospital on different occasions for the same cause or for various reasons in the same period. In order to capture this phenomenon, we used also the reported number of hospitalizations from the DGIS database.[26]

For Mexico, the Population Council of Mexico (CONAPO) provided the population projections data for different calendar years.[29] The mortality data for the study period come from the General Directorate of Health Information (DGIS).[30]

### Colombia data

We obtained the number of patients with IBD as primary diagnosis from the Integral Information System of Social Protection (SISPRO) database.[31] The SISPRO database, hosted by the Colombian Ministry of Health and Social Protection, offers consolidated data coming from all healthcare providers (hospitals and healthcare centers), which are obliged by law to report data using the ICD-10 coding for the primary diagnosis, for all registered persons who demand services within the Social Security Health System. It is considered that more than 90% of Colombians are registered with SISPRO.

SISPRO does not provide information on IBD patients not reaching the healthcare system, and thus there is no available data about them. To estimate the yearly incidence, we considered a “new case” to be a person with a positive diagnosis not previously identified, who required Health Services for the first time and is recorded as an IBD patient for the first time in SISPRO. Official demographic information and vital statistics were obtained from the National Administrative Department of Statistics (DANE). [32]

Prevalence of persons with IBD contacting the health system out of the total registered population was used as a proxy of real prevalence. Incidence per 100.000 population-years was based on the annual frequency of new IBD cases contacting the health system. These contacts could be either emergency visits, ambulatory consultations and/or hospital discharges.

### Modelling

We also used *mathematical modelling* to estimate the incidence and complete prevalence. DisMod II model is a multistate life table that simulates the epidemiological progress of a single disease by exploiting the fact that parameters such as incidence, prevalence, remission, case fatality and mortality rates are not independent variables.[33] Hence, it was designed to estimate epidemiological parameters on diseases when the availability of measured data is scarce. We used the Mexican and Colombian prevalence of the Institute of Health Metrics’ Global Burden of Disease project, estimated with Disease Modeling Multiregression (DISMOD-MR) as our input for the DISMOD II model for the years 2010 and 2016, for which data was available.[34]^,^[35] We assumed zero remission rate.

We also ran MIAMOD, a model originally developed for cancer complete prevalence estimates[36,37]. It uses a back-calculation method that could be applied to estimate the incidence and complete prevalence of IBD, as a chronic condition, from specific age-gender mortality and survival as inputs.[37] Mortality came from DGIS, and survival was extrapolated from Danish IBD cases, being Denmark a country with extensive high-quality surveillance information. Regarding the degree of coverage of mortality data we used WHO’s Global Health Observatory.[38] Mortality input values for models were adjusted for this type of under-reporting, which was 5.5% for Mexico and 6% for Colombia, according to UN tables.[38] In order to estimate complete prevalence figures with MIAMOD we performed a sensitivity analysis considering an additional correction of 10%, due to garbage coding, anticipating that this is the model that provides the upper limit estimations.

### Cases-series

Regarding clinical phenotypes of disease, during 2018 we chose two centers: Hospital Dr. Bernardo Sepúlveda, Colorectal surgery Department, in Mexico City, Mexico; and Clínica Universitaria Colombia–EPS Sánitas in Bogotá, Colombia. Those centers collected retrospectively a *case-series* of patients under clinical follow-up. These large referral centers included both outpatients and inpatients, and the diagnosis of IBD was established according to the most updated diagnostic recommendations for IBD. This included clinical features, endoscopy of the colon, other biochemical and imaging procedures like video capsule or pan-endoscopy, and biopsy. The date of IBD diagnosis was considered the starting point of the disease follow-up, and the symptoms onset date was recorded separately. We gathered demographic data of patients, family history of IBD, disease anatomical extension, severity of disease at the time of diagnosis, signs and symptoms, hospitalization episodes, relapse dates, and treatment patterns.

We **used** a sample of 200 consecutive cases from the routine database of the clinic cases that allow ±5% of semi-amplitude of the 95% confidence interval for the proportions of those least frequent symptoms. Univariate statistical analyses were performed to outline the study population. Wilcoxon test and X^2^ tests were done to determine differences in continuous and categorical variables, respectively. Dependent variable, UC clinical remission, was analyzed according to the main variables collected, through a multivariable regression analysis. We presented central estimations with their 95% confidence Intervals (CI), both in tables and in graphical format. We performed these analyses using Stata statistical software (v. 14.1, StataCorp, College Station, TX).

The study was approved by the Ethics Committee of the Instituto Mexicano del Seguro Social (IMSS), Ref. 09-B5-61-2800/201800 and **by** the Research Ethics Committee of the Fundación Universitaria Sanitas, Bogotá Colombia, Act 017–18.

## Results

### Epidemiology in Mexico

Data from Mexican administrative databases allowed us to present discharge rates by IBD but did not allow us to estimate incidence or prevalence of disease. In **S1 and S2 Files,** we present the overall and by-age group rates per 100,000 population of hospital discharges by IBD type and sex, and the discharge rates by age groups from 2010 to 2015 to complement our epidemiological estimates based on SINAIS data.

We present the results using mathematical modelling (DISMOD II AND MIAMOD) to estimate the incidence and complete prevalence.

The age-adjusted complete prevalence of UC per 100,000 inhabitants for 2015/2016 ranged from 15.65 to 71.19, depending on the model considered. The prevalence of CD per 100,000 inhabitants, ranged from 15.45 to 18.08. The age-adjusted incidence of UC per 100,000 inhabitants per year, ranged from 0.90 to 2.30, and for CD ranged from 0.35 to 0.66 using both models. The prevalence by sex for 2010 and 2015/2016 **is** presented in **Table 1**.

**Table 1:** Mexico 2010–16. Comparison of complete population prevalence and incidence rates of total IBD, as modelled by DisMod II and MIAMOD for the years 2010 and 2015/16.

Outcomes	Years	Ulcerative Colitis						Crohn's Disease					
		Male			Female			Male			Female		
		DisMod II^†^	MIAMOD		DisMod II^††^	MIAMOD		DisMod II^#^	MIAMOD		DisMod II^##^	MIAMOD	
			Base	+10%*		Base	+10%*		Base	+10%*		Base	+10%*
***Prevalence***	***2010***	19.20	39.80	43.90	17.79	84.08	98.30	21.16	18.15	20.02	16.71	14.63	16.09
	***2015/16***	17.00	39.84	43.95	14.3	84.20	98.43	20.50	18.16	20.05	10.40	14.65	16.11
***Incidence***	***2010***	1.00	1.41	1.55	0.68	2.65	3.05	0.30	0.68	0.75	0.30	0.52	0.57
	***2015/16***	1.00	1.41	1.55	0.80	2.65	3.05	0.30	0.68	0.75	0.40	0.52	0.57

Prevalence inputs based on DISMOD-MR [34]^,^[35]

^†^22 (2010) and 21 (2016)

^††^16 (2010) and 15 (2016)

^**#**^13 (2010) and 12 (2016)

^**##**^10 (2010) and 8.4 (2016).

*Results from MIAMOD with an adjusted of 10% in the specific mortality data; NE: Not Estimated.

The MIAMOD adjusted prevalence was twice as high as the DisMod II for men and six times the value for females, and regarding CD they were quite similar.

The MIAMOD adjusted was 1.5 and three times higher than the DisMod II in male and female UC incidence, respectively. We found similar female CD incidence and around two times higher for males (Table 1).

The female-male ratio for UC prevalence was 2.1: 1 and for the CD prevalence was 0.8:1 (MIAMOD) and 0.7:1 and 1:1 using DisMod II.

### Epidemiology in Colombia

We collected information of the years 2010–2015 from SISPRO, the most complete from the database inception. We found a total of 51,330 people who consulted with the Colombian Health System between 2010 and 2015. These people visited different centers whose data are submitted to the SISPRO registry. **Table 2** shows the information from this database, presenting the yearly number of people who have been admitted to hospitals, who have used the emergency services and who have received medical procedures due to inflammatory bowel disease at the outpatient setting.

**Table 2:** Colombia 2010–15. Number of hospital discharges, emergency and outpatient visits, by IBD type and sex (all ages).

Year	Ulcerative Colitis						Crohn's Disease					
	Males			Females			Males			Females		
	Hospital	Emerg.	Outp.	Hospital	Emerg.	Outp.	Hospital	Emerg.	Outp.	Hospital	Emerg.	Outp.
**2010**	137	129	3003	181	185	4166	20	25	487	10	23	801
**2011**	182	201	3639	252	266	4858	30	43	527	25	39	736
**2012**	211	280	4013	248	364	5355	39	53	526	38	65	643
**2013**	189	326	4190	217	395	5614	32	48	520	21	56	733
**2014**	224	385	4655	254	535	6291	43	58	603	32	67	796
**2015**	211	253	4124	268	342	5689	45	34	501	37	49	643

Source: SISPRO database

The frequency of drugs prescriptions in patients with IBD came from a sub-database of SISPRO including 837 patients who received specific IBD medication during the period 2010 to 2015. There was no data available for the calendar year 2012. About 72% (n = 599) received azathioprine while 56% (n = 469) and 36% (n = 305) received mesalazine and steroids, respectively.

According to our results, in Colombia the age-adjusted complete prevalence of UC per 100,000 inhabitants for 2015/2016 ranged from 27.40 to 69.97 depending on the model considered. The prevalence of CD per 100,000 inhabitants, ranged from 16.75 to 18.43. The age-adjusted incidence of UC per 100,000 inhabitants per year, ranged from 0.55 to 2.33, and for CD ranged from 0.30 to 0.57, respectively. These estimations were very similar to the 2010 estimations. The separate estimations by gender are presented in **Table 3**. The MIAMOD adjusted complete prevalence was 2 to 3 times higher than the DisMod II prevalence for UC and from 0.7 to 1.4 for CD. The MIAMOD adjusted incidence was 2 to 3 times higher than the DisMod II incidence for UC (although seven times higher for females) and from 1.6 to 2.3 times higher for CD. Regarding the comparison with SISPRO, the MIAMOD adjusted prevalence was 3 to 4 times higher than the cumulative proportion of UC patients registered in SISPRO, and 5 to 7 times higher for patients affected of CD.

**Table 3:** Comparison of complete population prevalence and incidence rates of UC and CD as modelled by DisMod II and MIAMOD for Colombia in the years 2010 and 2015/6, and from SISPRO.

Outcomes	Years	Ulcerative Colitis								Crohn's Disease							
		Male				Female				Male				Female			
		SISPRO	DisMod II^†^	MIAMOD		SISPRO	DisMod II^††^	MIAMOD		SISPRO	DisMod II^#^	MIAMOD		SISPRO	DisMod II^##^	MIAMOD	
				Base	+10%*			Base	+10%*			Base	+10%*			Base	+10%*
**Prevalence**	**2010**	16.76	16.57	47.79	52.36	21.8	19.24	79.27	87.2	2.73	19.29	12.89	14.17	4	15.38	20.58	22.64
	**2015/6**	20.25	26.6	47.85	54.3	26.72	28.2	79.39	87.33	2.56	20.2	12.9	14.19	3.1	16.6	20.61	22.68
**Incidence**	**2010**	13.7	0.81	1.69	1.86	18.4	0.48	2.52	2.81	2.6	0.31	0.44	0.48	3.9	0.29	0.61	0.7
	**2015/6**	13.5	0.7	1.69	1.86	18.8	0.4	2.48	2.81	1.6	0.3	0.43	0.48	2.1	0.3	0.60	0.7

Prevalence inputs based on DISMOD-MR34,35

†19 (2010) and 19 (2016)

††15 (2010) and 14 (2016)

#12 (2010) and 12 (2016)

##9.7 (2010) and 8.7 (2016).

*Results from MIAMOD with an adjusted of 10% in the specific mortality data; NE: Not Estimated.

In the **S2 File** we show the prevalence of IBD by sex and age group estimated using the MIAMOD model base case for both countries. The **S1 File** show the Colombian prevalence of UC and CD among health system users by age group from 2010 to 2015.

### Case-series

We recruited 404 IBD patients from Mexico D.F and Bogota City. The case-series in Mexico included a total of 200 patients with a diagnosis of IBD, 120 individuals had UC (60%) and 80 patients had CD (40%). The UC/CD ratio was 1.5: 1. The female-male ratio in UC was 1.5: 1 (60% vs 40%), and the CD ratio was 1.28: 1 (56.25% vs 47.75%). The Colombian case-series included a total of 204 patients with a diagnosis of IBD, 165 individuals had UC (80.88%), 36 patients had CD (17.65%) and 3 patients had an unclassifiable type of IBD (1.47%). The UC/CD ratio was 4.5:1. The female/male ratio in UC was 0.68:1 (40.61% vs 59.4%), and 0.8:1 (44.44% vs 55.56%) for CD. See **Table 4**.

**Table 4:** Clinical characteristics of UC and CD patients in the case-series of patients in Mexico City (n = 200) and Bogota (n = 204).

	Mexico			Colombia			
Characteristics	Ulcerative Colitis (n = 120)	Crohn's Disease (n = 80)	p	Ulcerative Colitis (n = 165)	Crohn's Disease (n = 36)	Non-specific (n = 3)	p
Women n (%)	72 (60)	45 (56.25)	0.59*	67 (40.6)	16 (44.4)	2 (66.7)	0.62*
Ever smoker (%)	50 (41.6)	42 (52.5)	0.44*	35 (21)	8 (22)	-	0.55*
Age at diagnosis—median (min-max)	40 (28.5–49.5)	49.5 (30.5–61.5)	0.03^#^	38 (12–80)	49 (12–85)	51 (25–68)	0.14^#^
Symptoms starting age—median (min-max)	38 (26.5–49)	49 (29.5–60)	0.01^#^	37 (11–80)	47 (11–84)	51 (23–68)	0.17^#^
Time to diagnosis—median years (min-max)	0 (0–1)	0.5 (0–2)	0.1^#^	0 (0–11)	0.5 (0–2)	-	0.39^#^

* X^2^ tests

^#^ Test U of Mann-Whitney.

**Table 5** shows the most frequent clinical manifestations in IBD patients in both countries. The most frequent symptoms in both case-series were diarrhea, rectal bleeding and abdominal pain. **Table 6** shows the prevalence ratio of the clinical manifestations in IBD patients of both cases-series. In Mexico, anorexia and weight loss were the clinical features most significantly related with UC, while abdominal pain was the symptom most frequently associated with CD. In relation with Colombian patients, bleeding was the clinical feature most significantly related with UC, while weight loss, abdominal pain, bowel obstruction and arthritis were the signs/symptoms most associated with CD. Diarrhea is equally prevalent in both pathologies.

**Table 5:** Clinical signs and symptoms observed in IBD case-series of patients in both countries.

	Mexico (n = 200)			Colombia (n = 201)		
Clinical signs and symptoms	Ulcerative Colitis (n = 120)	Crohn’s Disease (n = 80)	p*	Ulcerative Colitis (n = 165)	Crohn’s Disease (n = 35)	p*
Diarrhea n(%)	118 (98.50)	72 (90.00)	0.01	151 (91.5)	31 (86.1)	0.85
Rectal bleeding n(%)	119 (99.20)	72 (90.00)	0.01	147 (89.1)	24 (67.7)	0.01
Abdominal Pain n(%)	92 (77.00)	32 (40.00)	0.06	127 (77)	32 (88.9)	0.1
Weight Loss n(%)	43 (35.83)	52 (65.00)	<0.01	79 (47.9)	23 (63.9)	0.08
Bowel obstruction n(%)	17 (14.16)	19 (23.75)	0.07	11 (6.7)	5 (13.9)	0.05
Anorexia n(%)	24 (20.00)	39 (48.75)	<0.01	42 (25.4)	13 (36.1)	0.04
Fatigue n(%)	37 (30.83)	45 (56.25)	<0.01	8 (4.8)	4 (11.1)	0.23
Others symptoms n(%)	2 (1.74)	11 (13.75)	0.01	6 (3.6)	0	0.5
Arthritis/arthralgias n(%)	66 (55.00)	45 (56.25)	0.76	9 (5.4)	5 (13.9)	0.05

* X^2^ tests

**Table 6:** Prevalence ratio of selected clinical manifestations in UC versus CD patients, by country (2018).

	Mexico			Colombia		
Variable	Prevalence ratio UC* vs CD^§^	IC 95%	p	Prevalence ratio UC* vs CD^§^	IC 95%	p
Diarrhea	0.97	0.82–1.13	0.67	1.07	0.75–1.52	0.67
Bleeding	1.04	0.89–1.22	0.58	2.07	1.05–4.05	<0.01
Abdominal Pain	0.81	0.69–0.94	<0.01	0.79	0.73–0.86	0.04
Weight Loss	1.32	1.03–1.69	0.02	0.81	0.72–0.92	<0.01
Bowel obstruction	1.27	0.88–1.82	0.15	0.79	0.56–1.11	0.06
Anorexia	1.57	1.12–2.20	<0.01	0.85	0.75–1.03	0.06
Arthritis	1.01	0.83–1.22	0.92	0.77	0.52–1.14	0.07

*Ulcerative Colitis

^§^Crohn’s disease

Among Mexican patients, the most frequent anatomical involvement in CD patients were ileocolonic (37%) and colonic (30%) while the most frequent anatomical localization of UC patients was pancolitis with 55.83%, while left-sided colitis and proctitis were 34.17% and 10%, respectively. In Colombia, the most frequent anatomical involvement in CD patients were ileo-terminal (55%) and ileocolonic (39%), while the most frequent anatomical localization of UC patients was pancolitis with 39%, left-sided colitis 33.5% and proctitis 27.5%.

At time of data collection, the most frequent clinical condition in Mexican UC patients was clinical remission (83%), followed by mild and moderate compromise (14% and 3% respectively). Out of 102 of patients in remission, 100 presented clinical remission, 68 biochemical remissions, 63 presented endoscopic remission and 28 patients presented histopathological remission. In Colombia, 58% of UC patients presented clinical remission, 26% being mild, and 16% moderate in the category of severity.

In the Mexican case-series, the relapse rate of the IBD patients was 67% (134/200). The UC relapse rate was 63.33% (n = 76), while the CD relapse rate was 72.5% (n = 58) without statistics difference (p = 0.15). In comparison, in the Colombian case-series the relapse rate for IBD was 57.4% (117/204); 58.2% in UC patients (96/165), and 58.3% in CD ones (21/36).

In the UC group, 27.63% (21/76) of the Mexican patients presented the relapse between the first five years after diagnosis and the same number of patients relapsed after five years (21/76). Regarding the CD patients who presented relapses, 31.03% (18/58) and 22.41% (13/58) relapsed during the first five years and after the fifth year from diagnosis, respectively.

In Colombia, for those 96 UC patients who relapsed, 23% did so during the first 12 months after diagnosis, 53% between one year and five years after diagnosis, and 22% after five years, with 2% of patients with missing information. For those 21 CD patients who relapsed, 24% did in the first 12 months after diagnosis, 62% within the first to fifth year and the remaining 14%, after 5 years to diagnosis.

In Mexican case-series, 38% of UC cases (n = 46) and 72.5% of CD ones (n = 59) required hospitalization. The median number of hospitalizations in patients with UC was 1 (min-max: 1–4) and 2 (min-max: 1–15) in CD patients. The percentage of UC patients who required surgery during the follow-up was 10% (n = 12), contrasting with CD patients, who needed a surgery in 58.75% (n = 47) of cases.

If we consider the Colombian case series, 27% of UC cases (n = 45) and 36% of CD ones (n = 13) required hospitalization. The median number of hospitalizations in patients with ulcerative colitis was 1 (min-max: 1–8) while the median number of hospitalizations in patients with CD was 2 (min-max: 1–11). The percentage of UC patients who required surgery during the follow-up was 2% (n = 3), contrasting with CD patients, who needed surgery in 28% (n = 10) of cases.

Regarding the treatment pattern distribution, in Mexican UC case-series, 5-aminosalicylic acid (5-ASA) was the most frequent choice (98%), followed by steroids (48%), azathioprine (47%) and biologicals (22%); and among the CD patients, the most frequent were 5-ASA (98%), azathioprine (76%), biologicals (76%) and steroids (64%). In Colombia, 97% of UC patients received 5-ASA, and 56%, 51% and 20% used steroids, azathioprine and biological drugs, at any time during follow-up, respectively. For CD patients, the most frequent were steroids (86%), followed by azathioprine (84%), biologicals (64%) and finally 5-ASA (39%).

## Discussion

This work presented novel estimates of IBD disease burden and clinical features of affected patients in two Latin American countries, Mexico and Colombia. Given the lack of information on nationwide prevalence and incidence of IBD, we estimated them through different mathematical models and provided supplementary information coming from large official health system databases. To our knowledge, this is the first study doing so in both countries. In the case of Mexico, the official health system databases do not report persons with hospitalizations related to IBD. In Colombia, on the other hand, the cumulative proportion of IBD patients registered in SISPRO represents a good approximation of the country prevalence considering that most cases require the health system intervention and that the database is updated annually.

When we first analyzed the SISPRO incidence, we expected the biggest incidence rate to be for 2010, but we obtained similar values for incidence over the years that we included in the analysis. We consider two situations that may impact on the number of cases that have been registered in SISPRO: 1) the uploading of the cases depends on whether the administrative employees and physicians do that; and 2) the coverage of SISPRO has been growing in the last years and it is possible that each year different health centers are incorporated to the registry.

For Mexico, our study estimated a UC prevalence of 71 per 100,000 inhabitants (44 for males and up to 98 for females) in 2015/16; and of 18 for CD, (20 for males and up to 16 for females). The UC incidence per 100,000 inhabitants was 2.30 (1.55 for males and up to 3.05 for females), and for CD, it was 0.66 (0.75 and up to 0.57 for males and females respectively).

For Colombia, the UC prevalence was 70.5 per 100,000 inhabitants (54 for males and up to 87 for females in 2015/6); and for CD it was 21.5 per 100,000 inhabitants (20 for males and up to 23 for females). The UC incidence per 100,000 inhabitants was 2.3 per 100,000 inhabitants (1.86 for males and up to 2.81 for females), and for CD it was 0.59 per 100,000 inhabitants (0.48 for males and up to 0.7 for females).

The IBD prevalence figures obtained with MIAMOD for Mexico and Colombia were higher than those reported in a previous systematic review carried out by our research team in Latin American countries, in which the prevalence for UC ranged from 0.99 to 44.3 per 100,000 inhabitants, and from 0.24 to 14.90 for CD.[18]

IBD epidemiology has been studied in developed countries in a more comprehensive way, as noted by Ng et al in a study showing a rising incidence in industrialized countries in Africa, Asia, and South America since 1990.[8] The highest reported prevalence rates for IBD were in Europe (UC, 505 per 100,000 persons; CD, 322 per 100,000 persons) and in North America (UC, 286 per 100,000 persons in USA; CD, 319 per 100,000 persons in Canada).[9,10] Differences between countries could be explained by several factors, including migration rates, genetic profiles, environment or socioeconomic factors, and also by methodological limitations of epidemiologic studies, including design or diagnostic approach, differences in population characteristics, and access to health care.[39]^,^[40] Of note, all of those prevalence and incidence rates are much higher than those coming from a few well-conducted Latin American population-based studies.[18]

A study has revealed a decreasing geographical North-South gradient for IBD incidence. One possible explanation would be that in northern regions, there is low exposure to sunlight, as well as vitamin D plasma levels.[41] However, its causal relationship with IBD is still uncertain. The lower IBD prevalence in Mexico and Colombia compared to other countries in North America could also be due to their latitude, but considering the absence of population-based prevalence studies this theory deserves further research.

The varying range of our estimations can be partly explained by structural differences between models. DisMod II is a state-transition model which requires the population structure, general and specific mortality, remission and prevalence as input.[33] On the other hand, MIAMOD is a model of multiple and polynomial regressions that uses all-cause mortality, specific mortality, relative survival and population structure as input. As stated, MIAMOD model is a method primarily developed for cancers with long survival, to be used in countries with a sound surveillance system capable of adequately capturing survival rates. It has been used for colon and breast cancer and could potentially be applied to other chronic conditions like IBD.[36,37] Also as there might be some concerns regarding the quality of the survival data used for feeding MIAMOD, we decided to use the 14-year Denmark’s nationwide cohort, as the best available surrogate input of the age-adjusted survival.[42] Although it can be argued that IBD patients survival in a low- and middle-income country could be lower than **in** a high-income country, the magnitude of the difference would be minor since IBD mortality is low.[18] In general, MIAMOD prevalence and incidence estimates were higher than DisMod II model estimations.

The DisMod II model estimations and SISPRO information were quite similar regarding prevalence. Yepes Barreto et al in 2010 published a small case-series of adult population from a social security institution in Cartagena, and estimated an overall IBD prevalence in their population of 29 per 100,000 (95%CI 17–40), which is in the range of our estimations.[43]

Additionally, another source reinforced our estimations for prevalence. In 2016, the Health Metrics and Evaluation (IHME) published the updated Global Burden of Disease (GBD) for prevalence and incidence in Mexico and Colombia, in which they used the DisMod-MR model. This model is based on Bayesian meta-regression and uses an extensive temporal series of population surveys as input.[34]^,^[35] For Mexico, the GBD study estimated a 2016 UC prevalence per 100,000 inhabitants of 22 for males and 16 for females. For CD, it estimated a prevalence per 100,000 inhabitants of 12 and 9 for males and females, respectively. For Colombia, the GBD study estimated a 2016 UC prevalence per 100,000 inhabitants of 22 for males and 17 for females. For CD, it estimated a prevalence per 100,000 inhabitants of 12 and 9.4 for males and females, respectively. However, we identified some inconsistencies in the GBD reported incidence data for both countries, like the finding of higher incidence than prevalence rates.

We also analyzed the UC and CD GBD prevalence compared with the best quality population-based nationwide studies.[8] With the exception of two cases with just a one-digit prevalence (Romania and Taiwan), in all cases (Hungary, Japan, Barbados, Sweden, Germany, Denmark, Canada, Norway and Finland) GBD largely under-estimated the real prevalence. For this reason, we consider that our DisMod II estimations are conservative and the real prevalence could probably be higher and closer to our MIAMOD-derived estimations.

Our study had several limitations related mainly to the quality of available data and many assumptions required modelling the IBD incidence and prevalence by these novel methods. For example, the exercise of MIAMOD outside the field of oncology, with information only for a relatively small number of years and using foreign survival data, may threaten the validity of its estimates. Additionally, since the relative survival rates were not available, we used the observed absolute survival rates that could result in underestimation of the real prevalence rates.

On the other hand, the multiple methodological and epidemiological verification sources used to increase the confidence in our estimations represent a clear strength of our study. In Colombia, SISPRO databases also provided further valuable information like the number of hospital discharges, emergency and outpatient visits and treatment pattern by IBD type and by sex for all ages. A limitation of this database is that if a patient does not contact the health system, then that IBD case is ignored.

We found similar results in other case-series in Mexico and Colombia but with some differences. [44–47]

A recent multicentric nationwide study evaluated the clinical and socio-demographical characteristics of 2645 Mexican IBD patients from February to October 2017, with results that are consistent with our case-series.

Regarding the description of clinical phenotypes of Colombian IBD cases, Baños et al also reported a case-series of 202 patients drawn from a convenience sample in Medellín, Colombia. IBD patients (CD 15.8%, UC 80.7%) who consulted the reference center Hospital Pablo Tobón Uribe between August 2001 and July 2009 were systematically evaluated.[46] A slight predominance in the female group (56.4%) and an average age of onset of 37 years of age was found. The percentage of patients with extra-intestinal involvement was 27.7% and the time of diagnosis from the onset of symptoms was 9.2 months for UC and 13.2 for CD. The 35% of patients affected with UC presented extensive colitis and only 23.1% of them showed severe symptoms. The anatomical compromise of the patients with CD was predominantly ileocolonic (50%) and the inflammatory, stenotic and perianal behavior was of similar proportions in this group. The proportion of patients with UC who were in clinical remission was higher than that published by Baños (58.7% vs 17.2%) while the proportion of patients with mild UC was similar for both series of cases, probably due to newer available treatments.[46] Consistently, the proportion of patients with UC who had been hospitalized was 30.4% and 58.1% for CD while the value for the other case -series was 42.9% and 75% respectively.[46] Biological therapy was used in 7.4% of patients with UC and in 47% of patients with CD and its use was related to the risk of requiring surgical treatment in the two diseases.[48]

A previous large case-series of 848 patients with UC in Mexico reported by Yamamoto-Furusho in 2009[20] showed that the largest proportion of patients diagnosed with this pathology is between 21 and 30 years of age. The mean age at diagnosis was 31.3 years, while the median age was 38 years for patients with UC, lower than the ones in our study. The main clinical manifestations such as diarrhea, bleeding and abdominal pain were consistent with our case-series, as was the percentage of patients who required surgery (10%).[20] In relation with the distribution of the extent of the UC, it was consistent with our study. The comparative treatment pattern varied between our more recent series and the Yamamoto series: 5 ASA (98 vs. 64%), steroids (48 vs. 33%), azathioprine (47 vs 28%) and biologicals (22 vs 1%), respectively.[20] Another small case-series presented similar clinical manifestations and surgery requirements but different treatment patterns: 5 ASA (100%), steroids (7%), and biologicals (7%).[24] Other case-series of 104 patients with IBD showed that out of the 75 patients with UC, 55% had mild disease, 30% moderate disease and 15% severe disease.[23] Another study by Yamamoto-Furusho et al found that elderly patients with CD had a mild clinical course characterized by long-term remission and no escalation to immunosuppressive and anti-tumor necrosis factor therapy.[49]

Of interest, a cohort analysis of 184 cases in Mexico found that pancolitis, hypoalbuminemia and previous hospitalizations were the strongest predictors of procto-colectomy.[50]One study found a higher prevalence of left-sided colitis, higher mortality, and higher usage of biological agents in Mexican population than in Mexican-American population, supporting the role of environmental factors on clinical characteristics of IBD.[24]

The larger case-series in LAC Countries are from 1946 to date are from Argentina[14,51,52], Barbados[13], Brazil[14,53–65], Chile[66–68], Cuba[69], Peru[70,71], Puerto Rico[72–75] and Uruguay.[76–78] Most CD localizations were ileal-colonic and CD behavior was mostly inflammatory, followed by penetrant or stenotic. Perianal disease was identified roughly in one out of four cases. Regarding UC extension, it varied a lot, but extensive colitis was the most prevalent in these cases series, followed by left-sided and proctitis.

## Conclusion

Despite the aforementioned difficulties in approaching real IBD estimations in a scenario of paucity of representative data, we showed that the burden of IBD in Mexico and Colombia should not be considered negligible. Moreover, given the high variability observed in the reporting of IBD epidemiology worldwide, depicting real-world burden of these conditions remains to be a global challenge.

## Supporting information

S1 File Figures 1 to 4. (DOCX)

S2 File Tables 1 to 6. (DOCX)
