# Supplementary material for: Epidemiology of inflammatory bowel disease in Mexico and Colombia: Analysis of health databases, mathematical modelling and a case-series study
Source: PLoS One. 2020 Jan 27;15(1):e0228256. doi: 10.1371/journal.pone.0228256 (PMC6984728; doi:10.1371/journal.pone.0228256)
Supplement: S1 File — (DOCX) [file pone.0228256.s001.docx]

**Supplementary figures**

**S1** [**Fig. Ulcerative Colitis discharge rate by age group 2010-2015**](#FIG_6)**. SINAIS-Mexico**

**S2** **Fig. Crohn’s disease discharge rate by age group 2010-2015. SINAIS-Mexico**

**S3** **Fig. Prevalence of Crohn’s Disease among health system users* by age group from 2010 to 2015. SISPRO-Colombia**

* Hospital discharge + Emergency visits + Outpatients visits

**S4** **Fig. Prevalence of Ulcerative Colitis among health system users* by age group from 2010 to 2015. SISPRO-Colombia**

* Hospital discharge + Emergency visits + Outpatients visits
