# Supplementary material for: Epidemiology of inflammatory bowel disease in Mexico and Colombia: Analysis of health databases, mathematical modelling and a case-series study
Source: PLoS One. 2020 Jan 27;15(1):e0228256. doi: 10.1371/journal.pone.0228256 (PMC6984728; doi:10.1371/journal.pone.0228256)
Supplement: S2 File — (DOCX) [file pone.0228256.s002.docx]

**Supplementary tables**

**Table 1. SINAIS** **overall rates per 100,000 population of hospital discharges by IBD type and sex,** **all ages considered. (Mexico)**

| **Year** | **Ulcerative Colitis** | | | | | | **Crohn's Disease** | | | | | | |
| --- | --- | --- | --- | --- | --- | --- | --- | --- | --- | --- | --- | --- | --- |
|  | **Males** | | | **Females** | | | **Males** | | | | **Females** | | |
|  | **N** | **Rate*** | **(95% CI)** | **N** | **Rate*** | **(95% CI)** | **N** | **Rate*** | | **(95% CI)** | **N** | **Rate*** | **(95% CI)** |
| **2010** | 704 | 1.26 | (1.17-1.36) | 727 | 1.24 | (1.16-1.34) | 186 | 0.33 | (0.29-0.38) | | 219 | 0.37 | (0.33-0.43) |
| **2011** | 769 | 1.36 | (1.27-1.46) | 776 | 1.31 | (1.22-1.41) | 265 | 0.47 | (0.42-0.53) | | 244 | 0.41 | (0.36-0.47) |
| **2012** | 740 | 1.29 | (1.2-1.39) | 775 | 1.29 | (1.21-1.39) | 255 | 0.45 | (0.39-0.5) | | 264 | 0.44 | (0.39-0.5) |
| **2013** | 829 | 1.43 | (1.34-1.53) | 892 | 1.47 | (1.38-1.57) | 276 | 0.48 | (0.42-0.54) | | 258 | 0.43 | (0.38-0.48) |
| **2014** | 905 | 1.55 | (1.45-1.65) | 887 | 1.45 | (1.36-1.55) | 294 | 0.5 | (0.45-0.56) | | 263 | 0.43 | (0.38-0.48) |
| **2015** | 835 | 1.42 | (1.32-1.52) | 899 | 1.45 | (1.36-1.55) | 281 | 0.48 | (0.42-0.53) | | 310 | 0.5 | (0.45-0.56) |

**Table 2. SINAIS rates per 100,000 population of hospital discharges by IBD type, age group and sex. (Mexico)**

| **Year** | **Age** | **Ulcerative Colitis** | | | | **Crohn's Disease** | | | | | |
| --- | --- | --- | --- | --- | --- | --- | --- | --- | --- | --- | --- |
|  |  | **Males** | | **Females** | | **Males** | | | **Females** | | |
|  |  | **Rate*** | **(95% CI)** | **Rate*** | **(95% CI)** | **Rate*** | | **(95% CI)** | **Rate*** | | **(95% CI)** |
| **2010** | 0-14 | 0.38 | (0.30-0.49) | 0.41 | (0.32-0.52) | 0.15 | (0.10-0.22) | | 0.08 | | (0.05-0.14) |
|  | 15-24 | 0.9 | (0.74-1.11) | 0.66 | (0.53-0.84) | 0.25 | (0.17-0.37) | | 0.33 | | (0.24-0.46) |
|  | 25-54 | 1.66 | (1.50-1.84) | 1.5 | (1.35-1.67) | 0.41 | (0.33-0.5) | | 0.31 | | (0.25-0.39) |
|  | 55-64 | 2.59 | (2.11-3.19) | 2.46 | (2.01-3.01) | 0.64 | (0.42-0.97) | | 0.95 | | (0.69-1.32) |
|  | 65+ | 3.04 | (2.5-3.7) | 3.71 | (3.14-4.37) | 0.77 | (0.52-1.13) | | 1.6 | | (1.25-2.06) |
| **2011** | 0-14 | 0.46 | (0.37-0.58) | 0.39 | (0.31-0.5) | 0.21 | (0.16-0.3) | | 0.15 | (0.1-0.22) | |
|  | 15-24 | 0.77 | (0.62-0.95) | 0.83 | (0.67-1.02) | 0.26 | (0.18-0.37) | | 0.29 | (0.2-0.41) | |
|  | 25-54 | 1.79 | (1.62-1.98) | 1.55 | (1.4-1.72) | 0.56 | (0.47-0.67) | | 0.42 | (0.34-0.51) | |
|  | 55-64 | 2.72 | (2.23-3.32) | 2.9 | (2.41-3.48) | 1.09 | (0.8-1.5) | | 0.97 | (0.7-1.33) | |
|  | 65+ | 3.58 | (3.00-4.28) | 3.44 | (2.91-4.07) | 1.19 | (0.88-1.63) | | 1.3 | (0.99-1.71) | |
| **2012** | 0-14 | 0.15 | (0.1-0.22) | 0.20 | (0.14-0.28) | 0.14 | (0.09-0.21) | | 0.15 | (0.1-0.22) | |
|  | 15-24 | 0.35 | (0.25-0.48) | 0.37 | (0.27-0.5) | 0.31 | (0.22-0.43) | | 0.27 | (0.19-0.38) | |
|  | 25-54 | 0.61 | (0.51-0.72) | 0.45 | (0.38-0.55) | 0.61 | (0.52-0.72) | | 0.48 | (0.4-0.57) | |
|  | 55-64 | 0.54 | (0.35-0.84) | 0.61 | (0.41-0.9) | 0.76 | (0.52-1.09) | | 1.12 | (0.84-1.5) | |
|  | 65+ | 0.58 | (0.38-0.90) | 1.06 | (0.79-1.43) | 1.01 | (0.73-1.41) | | 1.21 | (0.91-1.6) | |
| **2013** | 0-14 | 0.28 | (0.21-0.37) | 0.21 | (0.15-0.3) | 0.22 | (0.16-0.3) | | 0.16 | (0.11-0.24) | |
|  | 15-24 | 0.38 | (0.28-0.52) | 0.36 | (0.26-0.49) | 0.26 | (0.18-0.38) | | 0.28 | (0.19-0.39) | |
|  | 25-54 | 0.67 | (0.57-0.79) | 0.54 | (0.46-0.64) | 0.60 | (0.51-0.71) | | 0.44 | (0.36-0.53) | |
|  | 55-64 | 0.50 | (0.32-0.77) | 0.84 | (0.61-1.17) | 1.04 | (0.77-1.42) | | 0.85 | (0.61-1.17) | |
|  | 65+ | 1.15 | (0.85-1.57) | 1.10 | (0.82-1.46) | 0.99 | (0.71-1.37) | | 1.36 | (1.05-1.76) | |
| **2014** | 0-14 | 0.17 | (0.12-0.24) | 0.18 | (0.12-0.25) | 0.12 | (0.08-0.19) | | 0.12 | (0.08-0.19) | |
|  | 15-24 | 0.39 | (0.29-0.52) | 0.39 | (0.29-0.53) | 0.22 | (0.15-0.33) | | 0.22 | (0.15-0.33) | |
|  | 25-54 | 0.71 | (0.61-0.83) | 0.5 | (0.42-0.60) | 0.67 | (0.57-0.78) | | 0.41 | (0.33-0.49) | |
|  | 55-64 | 0.38 | (0.23-0.62) | 0.68 | (0.47-0.97) | 1.21 | (0.91-1.60) | | 1.15 | (0.88-1.51) | |
|  | 65+ | 0.96 | (0.69-1.33) | 0.95 | (0.70-1.28) | 1.34 | (1.01-1.77) | | 1.52 | (1.20-1.94) | |
| **2015** | 0-14 | 0.10 | (0.06-0.16) | 0.10 | (0.06-0.17) | 0.15 | (0.10-0.22) | | 0.27 | (0.20-0.36) | |
|  | 15-24 | 0.36 | (0.26-0.49) | 0.35 | (0.25-0.48) | 0.25 | (0.17-0.36) | | 0.29 | (0.21-0.41) | |
|  | 25-54 | 0.43 | (0.36-0.52) | 0.43 | (0.36-0.52) | 0.59 | (0.50-0.70) | | 0.48 | (0.40- 0.57) | |
|  | 55-64 | 0.74 | (0.53-1.03) | 0.74 | (0.53-1.03) | 0.85 | (0.61-1.18) | | 1.04 | (0.79-1.38) | |
|  | 65+ | 0.98 | (0.73-1.32) | 0.98 | (0.73-1.32) | 1.51 | (1.16-1.95) | | 1.43 | (1.12-1.82) | |

**Table 3. Prevalence of ulcerative colitis per 100,000 inhabitants by age-group in Mexico by MIAMOD model**

| **Age Groups** | **2010** | | **2011** | | **2012** | | **2013** | | **2014** | | **2015** | |
| --- | --- | --- | --- | --- | --- | --- | --- | --- | --- | --- | --- | --- |
|  | **Men** | **Women** | **Men** | **Women** | **Men** | **Women** | **Men** | **Women** | **Men** | **Women** | **Men** | **Women** |
| **0** | 0.61 | 1.8 | 0.61 | 1.8 | 0.6 | 1.78 | 0.6 | 1.79 | 0.6 | 1.79 | 0.6 | 1.79 |
| **1 - 4** | 3.7 | 10.69 | 3.7 | 10.7 | 3.71 | 10.72 | 3.71 | 10.71 | 3.71 | 10.7 | 3.7 | 10.67 |
| **5 - 9** | 9.52 | 26.35 | 9.52 | 26.34 | 9.52 | 26.34 | 9.52 | 26.34 | 9.52 | 26.33 | 9.52 | 26.35 |
| **10 - 14** | 16.24 | 43.1 | 16.27 | 43.12 | 16.28 | 43.13 | 16.28 | 43.12 | 16.28 | 43.11 | 16.28 | 43.1 |
| **15 - 19** | 23.11 | 58.77 | 23.13 | 58.86 | 23.16 | 58.92 | 23.19 | 58.98 | 23.23 | 59.04 | 23.26 | 59.08 |
| **20 - 24** | 30.28 | 73.71 | 30.26 | 73.67 | 30.26 | 73.65 | 30.27 | 73.69 | 30.29 | 73.78 | 30.33 | 73.88 |
| **25 - 29** | 37.93 | 88.2 | 37.89 | 88.15 | 37.86 | 88.13 | 37.83 | 88.09 | 37.79 | 88.03 | 37.75 | 87.97 |
| **30 - 34** | 45.52 | 101.3 | 45.54 | 101.4 | 45.57 | 101.5 | 45.59 | 101.59 | 45.58 | 101.61 | 45.53 | 101.56 |
| **35 - 39** | 52.9 | 113.14 | 52.98 | 113.27 | 53.02 | 113.37 | 53.05 | 113.43 | 53.07 | 113.5 | 53.1 | 113.6 |
| **40 - 44** | 59.81 | 123.53 | 59.88 | 123.64 | 59.96 | 123.78 | 60.04 | 123.93 | 60.13 | 124.09 | 60.22 | 124.26 |
| **45 - 49** | 66.48 | 133.08 | 66.47 | 133.07 | 66.48 | 133.08 | 66.5 | 133.13 | 66.55 | 133.2 | 66.61 | 133.3 |
| **50 - 54** | 72.71 | 141.26 | 72.75 | 141.37 | 72.77 | 141.46 | 72.77 | 141.5 | 72.75 | 141.51 | 72.72 | 141.49 |
| **55 - 59** | 78.14 | 148.04 | 78.18 | 148.08 | 78.23 | 148.18 | 78.29 | 148.31 | 78.35 | 148.45 | 78.41 | 148.58 |
| **60 - 64** | 83.16 | 154.51 | 83.08 | 154.33 | 83.01 | 154.2 | 82.97 | 154.11 | 82.95 | 154.06 | 82.96 | 154.08 |
| **65 - 69** | 86.81 | 159.13 | 86.76 | 159.04 | 86.72 | 159 | 86.67 | 158.95 | 86.6 | 158.84 | 86.52 | 158.71 |
| **70 - 74** | 89.15 | 162.13 | 89.08 | 161.93 | 89 | 161.8 | 88.94 | 161.76 | 88.89 | 161.72 | 88.83 | 161.67 |
| **75 - 79** | 90.35 | 163.65 | 90.41 | 163.56 | 90.42 | 163.53 | 90.39 | 163.63 | 90.34 | 163.51 | 90.27 | 163.5 |
| **80 - 84** | 83.6 | 151.55 | 83.59 | 151.45 | 83.59 | 151.54 | 83.62 | 151.84 | 83.65 | 151.85 | 83.67 | 151.95 |

**Table 4. Prevalence of Crohn’s disease per 100,000 inhabitants by age-group in Mexico by MIAMOD model**

| **Age Groups** | **2010** | | **2011** | | **2012** | | **2013** | | **2014** | | **2015** | |
| --- | --- | --- | --- | --- | --- | --- | --- | --- | --- | --- | --- | --- |
|  | **Men** | **Women** | **Men** | **Women** | **Men** | **Women** | **Men** | **Women** | **Men** | **Women** | **Men** | **Women** |
| **0** | 0.16 | 0.27 | 0.16 | 0.27 | 0.15 | 0.26 | 0.15 | 0.26 | 0.15 | 0.26 | 0.15 | 0.26 |
| **1 - 4** | 0.99 | 1.59 | 0.99 | 1.6 | 1 | 1.6 | 1 | 1.6 | 1 | 1.6 | 0.99 | 1.59 |
| **5 - 9** | 2.79 | 3.97 | 2.79 | 3.97 | 2.79 | 3.97 | 2.79 | 3.97 | 2.79 | 3.97 | 2.79 | 3.97 |
| **10 - 14** | 5.23 | 6.58 | 5.24 | 6.58 | 5.24 | 6.58 | 5.24 | 6.58 | 5.24 | 6.58 | 5.24 | 6.58 |
| **15 - 19** | 8.15 | 9.1 | 8.15 | 9.11 | 8.16 | 9.12 | 8.17 | 9.13 | 8.19 | 9.14 | 8.2 | 9.15 |
| **20 - 24** | 11.59 | 11.62 | 11.58 | 11.62 | 11.58 | 11.61 | 11.59 | 11.62 | 11.6 | 11.63 | 11.61 | 11.65 |
| **25 - 29** | 15.63 | 14.21 | 15.61 | 14.2 | 15.6 | 14.2 | 15.59 | 14.19 | 15.57 | 14.18 | 15.55 | 14.17 |
| **30 - 34** | 19.98 | 16.7 | 19.99 | 16.72 | 20 | 16.73 | 20.01 | 16.75 | 20.01 | 16.75 | 19.98 | 16.74 |
| **35 - 39** | 24.46 | 19.14 | 24.5 | 19.16 | 24.52 | 19.18 | 24.53 | 19.19 | 24.54 | 19.2 | 24.56 | 19.22 |
| **40 - 44** | 28.78 | 21.45 | 28.81 | 21.47 | 28.85 | 21.49 | 28.9 | 21.52 | 28.94 | 21.55 | 28.98 | 21.58 |
| **45 - 49** | 32.87 | 23.7 | 32.86 | 23.69 | 32.87 | 23.69 | 32.88 | 23.7 | 32.9 | 23.72 | 32.93 | 23.73 |
| **50 - 54** | 36.54 | 25.79 | 36.56 | 25.81 | 36.57 | 25.83 | 36.57 | 25.83 | 36.56 | 25.84 | 36.54 | 25.83 |
| **55 - 59** | 39.58 | 27.74 | 39.6 | 27.75 | 39.63 | 27.77 | 39.66 | 27.79 | 39.69 | 27.82 | 39.72 | 27.84 |
| **60 - 64** | 42.04 | 29.67 | 42 | 29.64 | 41.96 | 29.61 | 41.94 | 29.59 | 41.93 | 29.58 | 41.94 | 29.59 |
| **65 - 69** | 43.49 | 31.19 | 43.47 | 31.18 | 43.45 | 31.17 | 43.43 | 31.16 | 43.39 | 31.13 | 43.35 | 31.11 |
| **70 - 74** | 44.24 | 32.4 | 44.2 | 32.36 | 44.17 | 32.33 | 44.14 | 32.32 | 44.11 | 32.31 | 44.08 | 32.3 |
| **75 - 79** | 44.47 | 33.46 | 44.49 | 33.44 | 44.5 | 33.44 | 44.48 | 33.46 | 44.46 | 33.43 | 44.43 | 33.43 |
| **80 - 84** | 40.9 | 31.54 | 40.9 | 31.52 | 40.9 | 31.53 | 40.91 | 31.6 | 40.93 | 31.6 | 40.94 | 31.62 |

**Table 5. Prevalence of Ulcerative Colitis per 100,000 inhabitants by age-group in Colombia by MIAMOD model.**

| **Age Groups** | **2010** | | **2011** | | **2012** | | **2013** | | **2014** | | **2015** | |
| --- | --- | --- | --- | --- | --- | --- | --- | --- | --- | --- | --- | --- |
|  | Men | Women | Men | Women | Men | Women | Men | Women | Men | Women | Men | Women |
| **0** | 0.86 | 1.69 | 0.86 | 1.69 | 0.86 | 1.69 | 0.86 | 1.69 | 0.86 | 1.69 | 0.86 | 1.69 |
| **1 - 4** | 5.17 | 10.04 | 5.16 | 10.03 | 5.16 | 10.02 | 5.16 | 10.01 | 5.15 | 10.01 | 5.15 | 10 |
| **5 - 9** | 12.94 | 24.72 | 12.93 | 24.7 | 12.92 | 24.67 | 12.9 | 24.65 | 12.89 | 24.62 | 12.88 | 24.6 |
| **10 - 14** | 21.4 | 40.1 | 21.42 | 40.13 | 21.42 | 40.15 | 21.41 | 40.15 | 21.4 | 40.15 | 21.39 | 40.13 |
| **15 - 19** | 29.54 | 54.38 | 29.6 | 54.45 | 29.64 | 54.53 | 29.69 | 54.62 | 29.73 | 54.69 | 29.76 | 54.75 |
| **20 - 24** | 37.48 | 68.03 | 37.52 | 68.05 | 37.58 | 68.09 | 37.66 | 68.13 | 37.73 | 68.19 | 37.81 | 68.26 |
| **25 - 29** | 45.71 | 81.09 | 45.67 | 81.13 | 45.66 | 81.14 | 45.64 | 81.14 | 45.63 | 81.14 | 45.66 | 81.16 |
| **30 - 34** | 53.88 | 93.25 | 53.84 | 93.23 | 53.84 | 93.27 | 53.85 | 93.36 | 53.86 | 93.45 | 53.86 | 93.51 |
| **35 - 39** | 62.57 | 105.93 | 62.46 | 105.74 | 62.32 | 105.51 | 62.19 | 105.26 | 62.07 | 105.07 | 62 | 104.96 |
| **40 - 44** | 70.71 | 116.94 | 70.77 | 117.07 | 70.78 | 117.14 | 70.75 | 117.15 | 70.68 | 117.08 | 70.58 | 116.94 |
| **45 - 49** | 77.46 | 125.54 | 77.65 | 125.83 | 77.85 | 126.16 | 78.06 | 126.48 | 78.23 | 126.77 | 78.36 | 126.98 |
| **50 - 54** | 84.11 | 133.38 | 84.17 | 133.52 | 84.26 | 133.69 | 84.38 | 133.91 | 84.52 | 134.16 | 84.71 | 134.45 |
| **55 - 59** | 91.01 | 141.23 | 91.01 | 141.23 | 91 | 141.25 | 90.98 | 141.27 | 90.99 | 141.33 | 91.04 | 141.45 |
| **60 - 64** | 97.19 | 148.04 | 97.18 | 148 | 97.22 | 148.06 | 97.27 | 148.12 | 97.3 | 148.19 | 97.32 | 148.23 |
| **65 - 69** | 102.75 | 154.46 | 102.47 | 153.99 | 102.28 | 153.64 | 102.16 | 153.4 | 102.12 | 153.31 | 102.16 | 153.3 |
| **70 - 74** | 106.8 | 158.11 | 106.75 | 158.18 | 106.75 | 158.21 | 106.64 | 158.04 | 106.43 | 157.79 | 106.24 | 157.42 |
| **75 - 79** | 113.11 | 165.49 | 113.05 | 165.34 | 113.14 | 165.42 | 113.22 | 165.54 | 113.35 | 165.86 | 113.46 | 166.16 |
| **80 - 84** | 108.51 | 155.17 | 108.4 | 154.97 | 108.49 | 154.99 | 108.57 | 155.08 | 108.9 | 155.52 | 109.22 | 155.85 |

**Table 6. Prevalence of Crohn's Disease per 100,000 inhabitants by age-group in Colombia by MIAMOD model.**

| **Age Groups** | **2010** | | **2011** | | **2012** | | **2013** | | **2014** | | **2015** | |
| --- | --- | --- | --- | --- | --- | --- | --- | --- | --- | --- | --- | --- |
|  | **Men** | **Women** | **Men** | **Women** | **Men** | **Women** | **Men** | **Women** | **Men** | **Women** | **Men** | **Women** |
| **0** | 0.27 | 0.56 | 0.27 | 0.56 | 0.27 | 0.56 | 0.27 | 0.56 | 0.27 | 0.56 | 0.27 | 0.56 |
| **1 - 4** | 1.59 | 3.26 | 1.59 | 3.25 | 1.59 | 3.25 | 1.59 | 3.25 | 1.59 | 3.25 | 1.59 | 3.25 |
| **5 - 9** | 3.91 | 7.76 | 3.91 | 7.75 | 3.91 | 7.74 | 3.9 | 7.74 | 3.9 | 7.73 | 3.9 | 7.72 |
| **10 - 14** | 6.35 | 12.13 | 6.36 | 12.14 | 6.36 | 12.14 | 6.36 | 12.14 | 6.35 | 12.14 | 6.35 | 12.14 |
| **15 - 19** | 8.63 | 15.89 | 8.65 | 15.91 | 8.66 | 15.93 | 8.67 | 15.95 | 8.68 | 15.97 | 8.69 | 15.99 |
| **20 - 24** | 10.77 | 19.25 | 10.78 | 19.26 | 10.8 | 19.27 | 10.82 | 19.28 | 10.85 | 19.29 | 10.87 | 19.32 |
| **25 - 29** | 12.93 | 22.28 | 12.92 | 22.29 | 12.91 | 22.29 | 12.91 | 22.29 | 12.91 | 22.29 | 12.91 | 22.3 |
| **30 - 34** | 15 | 24.94 | 14.99 | 24.93 | 14.99 | 24.94 | 14.99 | 24.97 | 14.99 | 24.99 | 14.99 | 25 |
| **35 - 39** | 17.15 | 27.62 | 17.12 | 27.57 | 17.08 | 27.51 | 17.04 | 27.45 | 17.01 | 27.4 | 16.99 | 27.37 |
| **40 - 44** | 19.07 | 29.78 | 19.09 | 29.81 | 19.09 | 29.83 | 19.08 | 29.83 | 19.06 | 29.81 | 19.04 | 29.78 |
| **45 - 49** | 20.56 | 31.26 | 20.61 | 31.33 | 20.67 | 31.41 | 20.72 | 31.49 | 20.77 | 31.56 | 20.8 | 31.61 |
| **50 - 54** | 21.97 | 32.51 | 21.98 | 32.55 | 22.01 | 32.59 | 22.04 | 32.64 | 22.08 | 32.7 | 22.12 | 32.77 |
| **55 - 59** | 23.4 | 33.75 | 23.4 | 33.75 | 23.4 | 33.76 | 23.39 | 33.76 | 23.4 | 33.78 | 23.41 | 33.81 |
| **60 - 64** | 24.58 | 34.76 | 24.57 | 34.75 | 24.58 | 34.76 | 24.6 | 34.78 | 24.6 | 34.79 | 24.61 | 34.8 |
| **65 - 69** | 25.58 | 35.75 | 25.51 | 35.64 | 25.46 | 35.56 | 25.43 | 35.5 | 25.42 | 35.48 | 25.43 | 35.48 |
| **70 - 74** | 26.29 | 36.23 | 26.27 | 36.24 | 26.27 | 36.25 | 26.25 | 36.21 | 26.19 | 36.16 | 26.15 | 36.07 |
| **75 - 79** | 27.62 | 37.78 | 27.61 | 37.75 | 27.63 | 37.76 | 27.65 | 37.79 | 27.68 | 37.87 | 27.7 | 37.93 |
| **80 - 84** | 26.37 | 35.4 | 26.34 | 35.35 | 26.36 | 35.35 | 26.38 | 35.37 | 26.46 | 35.48 | 26.54 | 35.55 |
